# Content congruency and its interplay with temporal synchrony modulate integration between rhythmic audiovisual streams

**DOI:** 10.3389/fnint.2014.00092

**Published:** 2014-12-08

**Authors:** Yi-Huang Su

**Affiliations:** Department of Movement Science, Faculty of Sport and Health Sciences, Technical University of MunichMunich, Germany

**Keywords:** multisensory integration, rhythm, content congruency, audiovisual synchrony, attention

## Abstract

Both lower-level stimulus factors (e.g., temporal proximity) and higher-level cognitive factors (e.g., content congruency) are known to influence multisensory integration. The former can direct attention in a converging manner, and the latter can indicate whether information from the two modalities belongs together. The present research investigated whether and how these two factors interacted in the perception of rhythmic, audiovisual (AV) streams derived from a human movement scenario. Congruency here was based on sensorimotor correspondence pertaining to rhythm perception. Participants attended to bimodal stimuli consisting of a humanlike figure moving regularly to a sequence of auditory beat, and detected a possible auditory temporal deviant. The figure moved either downwards (congruently) or upwards (incongruently) to the downbeat, while in both situations the movement was either synchronous with the beat, or lagging behind it. Greater cross-modal binding was expected to hinder deviant detection. Results revealed poorer detection for congruent than for incongruent streams, suggesting stronger integration in the former. False alarms increased in asynchronous stimuli only for congruent streams, indicating greater tendency for deviant report due to visual capture of asynchronous auditory events. In addition, a greater increase in perceived synchrony was associated with a greater reduction in false alarms for congruent streams, while the pattern was reversed for incongruent ones. These results demonstrate that content congruency as a top-down factor not only promotes integration, but also modulates bottom-up effects of synchrony. Results are also discussed regarding how theories of integration and attentional entrainment may be combined in the context of rhythmic multisensory stimuli.

## Introduction

A key function of the perceptual system is its ability to continuously track and integrate information originating from different sensory modalities. Previous investigations of multisensory integration, employing paradigms with relatively simple bimodal stimuli (Meredith and Stein, 1983; Alvarado et al., 2007; Stein and Stanford, 2008; Stevenson et al., 2014a), have identified several factors related to the stimulus features that mediate the integration process. Amongst the most robust findings is that temporal proximity between the bimodal events promotes cross-modal integration (Chen and Vroomen, 2013). Integration is typically shown as enhanced neuronal response as well as behavioral advantages to concurrent multisensory information, compared to those in the most effective unisensory situation. Findings along this line suggest that temporally convergent information directs (or “captures”) attention in a stimulus-driven, bottom-up manner (Van der Burg et al., 2008), which facilitates subsequent binding of the inter-sensory signals (Fiebelkorn et al., 2010; Koelewijn et al., 2010; Talsma et al., 2010).

Integration in more complex multisensory stimuli can also be modulated by aspects of higher-level stimulus content. One such factor that especially concerns the present research is content congruency, i.e., the perceived content match between the bimodal stimuli based on their semantic correspondence or consistency (Doehrmann and Naumer, 2008). Stimuli that are matched in content tend to be treated as originating from the same source, and are thus more likely to be integrated by the perceptual system—also referred to as the *unity assumption* (Welch and Warren, 1980). This has been demonstrated in audiovisual (AV) speech, in which integration is favored when the spoken sound matches the gender of the talking face (Vatakis and Spence, 2007), or when the spoken syllable matches the facial articulatory movement (van Wassenhove et al., 2007; Ten Oever et al., 2013), compared to when they mismatch. In non-speech AV human actions, stronger integration has been found for a drumming movement paired with congruent than with incongruent impact sounds (Arrighi et al., 2006; Petrini et al., 2009a). In a similar vein, effects of AV content congruency have also been shown in biological motion perception. In those studies, visual detection of a walking humanlike point-light figure, (“PLF”, Johansson, 1973; Blake and Shiffrar, 2007) embedded in random dots is enhanced if the accompanying sounds convey natural footstep information compared to artificial tones (Thomas and Shiffrar, 2010, 2013), or when the direction of the moving sounds matches that of the walking PLF (Brooks et al., 2007; Schouten et al., 2011; Wuerger et al., 2012). In the scenarios discussed thus far, perceived content congruency relies on various learned associations between the bimodal stimuli. Such prior knowledge represents a cognitive factor that modulates multisensory integration in a top-down manner, which may also interact with lower-level stimulus factors (e.g., temporal relation) in the perceptual decision (Ten Oever et al., 2013; Stevenson et al., 2014b). Similarly, while temporal alignment drives attention in a bottom-up manner for cross-modal binding (i.e., through attentional spread), highly learned associations between bimodal stimuli can additionally activate a top-down attentional mechanism for integration (Fiebelkorn et al., 2010).

Perhaps not surprisingly, both the speech and non-speech AV stimuli mentioned above involve human movements, in which the sounds are consequent upon the viewed actions. That is, the auditory and the visual information is causally linked. Thus, based on prior experiences, a perceiver will generate certain expectations upon stimulus presentation, which can be used for temporal prediction in the ongoing bimodal streams (Lee and Noppeney, 2014; van Atteveldt et al., 2014). For example, in natural AV speech, the lip movements and the spoken sounds are temporally correlated, and the former typically precedes the latter (Chandrasekaran et al., 2009). This makes it possible for an observer to use the visual cues to predict when the sounds should occur (van Wassenhove et al., 2005; Zion Golumbic et al., 2013), by which attention can be directed to the expected points in time to support auditory processing (Lakatos et al., 2008) and, eventually, multisensory integration (van Atteveldt et al., 2014). Similarly, in non-speech AV actions such as drumming, the trajectory of the arm movement predicts the temporal occurrence of the impact sounds. The availability of visual movement cues for cross-modal prediction is also found to affect the strength of integration in this scenario (Arrighi et al., 2006; Petrini et al., 2009b). Notably, the predictive mechanism can be influenced by cognitive factors such as content congruency. Streams matched in content tend to be attributed to the same source of action, which then increases the likelihood that a perceiver would use cues in one modality to predict event occurrences in the other modality.

Given the role of the stimulus (temporal) and the cognitive factors, as well as the predictive mechanism in multisensory integration, one question may arise from here. In the course of AV action perception, besides the cross-modal prediction that is perpetuated by the stimulus correlation and the content match, there exists a possibility of temporal prediction within each modality. This may be especially true for bimodal stimuli that yield a perceivable periodicity in both sensory streams. The most prominent examples are rhythmic human movements that produce rhythmic sounds, e.g., drumming (Arrighi et al., 2006; Petrini et al., 2009b), hand clapping (Sevdalis and Keller, 2010), or walking (Thomas and Shiffrar, 2010, 2013). Speech, albeit with temporal variations, is also rhythmic along various time scales (Rothermich et al., 2012; Ghazanfar, 2013; Patel, 2014). For each modality, the underlying periodicity in the rhythmic stimulus can entrain attention accordingly, leading the perceiver to generate expectations/predictions of event occurrences at regular points in time (*Dynamic Attending Theory*, “DAT”, Large and Jones, 1999). As a result, stimulus processing is enhanced at these expected moments. This has been most frequently reported in the auditory modality (Jones et al., 2002; Large and Snyder, 2009; Repp, 2010); however, recent studies demonstrate that temporal entrainment can occur cross-modally, such that attention entrained by auditory rhythms can facilitate visual processing (Bolger et al., 2013, 2014), and the other way around (Su, 2014a). As such, in the course of multisensory perception of rhythmic human movements, both within-modal and cross-modal predictions may occur, and both mechanisms can deploy attention to convergent points in time that in turn promotes integration. Because integration is often measured by tasks that require judging the relation between both streams, i.e., synchrony judgment (SJ) or temporal order judgment (TOJ; Vroomen and Keetels, 2010), it is difficult to disentangle these two modes of prediction. It thus remains unclear to what extent each prediction mode contributes to the attentional deployment in multisensory perception, and whether either or both interact with other stimulus and cognitive factors.

Motivated by these issues, the present study set out to address several questions in multisensory perception involving continuous, rhythmic human movements. First, as opposed to causally linked AV actions, would the top-down effect of content congruency on integration be obtained in scenarios where the sounds are *not* caused by the movement, but rather that the movement is coordinated with extraneous sounds? The rationale behind was that content congruency can be based on various forms of association, and its effect has also been found for stimuli exhibiting abstract, synesthetic correspondences (Parise and Spence, 2009). In terms of humans moving along with sounds, such as dancing to music, a correspondence may exist as to which kind of movement is typically performed with regard to the rhythm of continuous sounds: For example, humans tend to move their body vertically to a musical beat (Toiviainen et al., 2010), and they most often move downwards rather than upwards to the beat (Miura et al., 2011; Su, 2014b). As no study has examined congruency regarding such action-perception association, this constituted the first question of interest in the present research. The next question asked whether, in this particular scenario, temporal proximity (i.e., synchrony) between the auditory and visual streams would also direct attention in a bottom-up manner to promote integration. More importantly, the focus was whether this stimulus-driven, temporal factor would interact with the cognitive factor of content congruency, which has recently been shown in AV perception of speech syllables (Ten Oever et al., 2013) but has not been investigated in a non-speech action domain. Finally, as both the auditory and visual streams were rhythmic in this case, it was of interest to examine whether within-modal or cross-modal predictive mechanism plays a dominant role when the task probes the perceptual outcome in one modality.

To this end, the present study employed an AV paradigm that resembled the scenario of observing a person moving to music. Here, a humanlike figure performed a whole-body bouncing movement vertically and periodically (as in Su, 2014a,c) to a sequence of regular auditory beat. The movement could be either congruent (moving *down* to the beat) or incongruent (moving *up* to the beat) to the auditory rhythm, and in both cases the movement could be either synchronous with the beat, or lagging behind the beat. Instead of a SJ or TOJ task, the present task required detection of a temporal deviant only in the auditory stream. Because the auditory sequence had a clear periodicity and the task was only auditory, there should be no effect of any of the visual manipulations if auditory prediction alone were adopted to perform the task. However, if the visual information were obligatorily incorporated into the auditory percept, i.e., if integration took place, then the AV streams should become temporally bound as a whole in perception. Consequently, one might become less sensitive to a slight deviation in one stream, resembling the reserved version of “temporal ventriloquism” (Fendrich and Corballis, 2001; Morein-Zamir et al., 2003). That is, the stronger the integration, the more the visual stream would temporally “capture” the auditory deviant, making it less salient than otherwise. As such, factors contributing to AV integration—synchrony, congruency, or both—should lead to *decreased* detection of the auditory deviant. Of interest, then, was whether synchrony and congruency operate independently, or whether they interact with each other in this process.

## Methods

### Participants

Fourteen paid volunteers (five male, mean age 27 years, SD = 6) participated in this experiment. All reported normal or corrected-to-normal vision and normal hearing. Participants were not pre-screened for musical training and varied in the length of training. The training duration ranged from 0–20 years (all amateur musicians), with a mean duration of 8 years (SD = 6). Amongst the amateur musicians (13), the learned instruments included piano or keyboard (10), percussion (2), and guitar (1). This study had been approved by the ethic commission of Technical University of Munich, and was conducted in accordance with the ethical standards of the 1964 Declaration of Helsinki. All participants gave written informed consent prior to the experiment.

### Stimuli and materials

**Figure 1:**
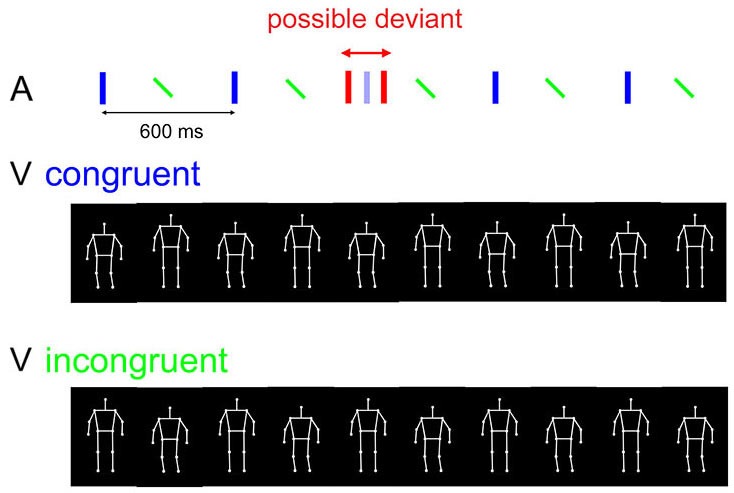
**Stimuli and trial procedure of the experiment.** In each trial, an auditory beat sequence (upper panel) consisted of five alternating downbeats (blue vertical bars) and upbeats (tilted green bars), where accents were perceived in the former. The beat sequence was combined with either a congruently moving PLF (middle panel; moving *down* to the downbeat) or an incongruently moving PLF (lowest panel; moving *up* to the downbeat). Red bars depict a potential auditory deviant, which could be a delayed or advanced downbeat relative to its original temporal position (shown in opaque blue). The manipulation of synchrony between the auditory and visual streams is not illustrated here.

***Visual Stimuli***. The visual stimuli consisted of a humanlike PLF performing a repetitive whole-body bouncing movement (i.e., repetitive knee flexion and extension), without the feet leaving the ground. The PLF was initially constructed by recording a practiced actor performing this movement continuously using a 3D motion capture system (Qualisys Oqus, 8 cameras), with a sampling rate of 200 Hz. 13 markers in total were attached to the major joints (Johansson, 1973). The recorded motion data were converted into a 2D (without depth information) point-light display in Matlab ®R2012b (Mathworks) using Psychophysics Toolbox extensions version 3 (Brainard, 1997), and the animation was down-sampled to 100 Hz to match the monitor’s frame frequency. The PLF was represented by 13 white discs against a black background, each of which subtended 0.4° of visual angle (°). In order to convey the human figure unambiguously, white lines were added to connect the discs^1^. The whole PLF subtended approximately 5° and 12° when viewed at 80 cm, and was centered in the middle of the screen (See also Figure 1 in Su (2014c)).

Each movement cycle consisted of a downward (knee flexion) and an upward (knee extension) phase. The former corresponded to 345 ms and the latter 255 ms on average across all the moving discs, as shown in the recorded motion data. The PLF movement was presented at a tempo corresponding to an inter-bounce interval of 600 ms, i.e., the temporal interval between the lowest positions (the “bounce”) of two consecutive cycles was 600 ms. Very similar visual stimuli were employed in three recent studies (Su, 2014a,b,c), in which steps of motion data processing and relevant parameters were described in detail. In Su (2014b), detailed information regarding the motion profile of the PLF movement can also be found. Here, as in Su (2014a,c), the PLF movement was presented as iterations of a single cycle. Slight temporal and spatial interpolations had been applied to the motion data to ensure that there was no temporal or spatial discrepancy when the movement was displayed cyclically.

***Auditory stimuli***. The auditory stimuli consisted of repetitive cycles of alternating “downbeat” and “upbeat” tones as employed in Su (2014b). The sounds were generated as wave files by the music software Logic 8 Express (Apple Inc. California). The downbeat tones had a synthesized sound of the instrument “bongo” with 50 ms tone duration, and the upbeat tones had a synthesized sound of the instrument “high hat” with 47 ms tone duration. The inter-downbeat interval was 600 ms, corresponding to a cycle of the PLF movement. To match the auditory temporal structure to the uneven movement phases of the PLF, the interval between a downbeat and its following upbeat was 255 ms/345 ms for stimuli in the AV congruent/incongruent conditions (see Section Procedure and Design). The downbeat tones had a lower timbre, and the upbeat tones were attenuated by 10 dB relative to the downbeat tones. As such, regular accents in the auditory sequence were unambiguously perceived at the downbeat positions (Su, 2014b).

### Procedure and design

The experimental program was controlled by a customized Matlab script using Psychophysics Toolbox version 3 routines running on a Mac OSX environment. The visual stimuli were displayed on a 17-inch CRT monitor (Fujitsu X178 P117A) with a frame frequency of 100 Hz at a spatial resolution of 1024 × 768 pixels. Participants sat with a viewing distance of 80 cm. Sounds were presented at a sampling rate of 44,100 Hz through closed studio headphones (AKG K271 MKII).

Each trial started with a fixation cross in the center of the screen for 1000 ms, followed by a presentation of five cycles of concurrent visual and auditory sequences. The visual sequence was a periodically bouncing PLF, and the auditory sequence consisted of repetitive downbeats and upbeats in alternation. The visual and auditory sequences were presented in four combinations that varied in terms of content congruency and temporal synchrony between the two streams. Content congruency was based on the correspondence between the movement phase and the auditory beat: In half of all the trials, the PLF was bouncing *downwards* to the downbeat (congruent); in the other half, the PLF was bouncing *upwards* to the downbeat (incongruent). In terms of synchrony, in half of all the trials the PLF moved synchronously to the auditory beat; namely, the lowest/highest position of the movement coincided with the auditory downbeat in the congruent/incongruent condition (as in (Su (2014a), Exp 2)). In the other half of the trials, the visual stream was phase shifted with a delay of 150 ms relative to the auditory stream. This lag was chosen on the basis of it slightly exceeding the temporal integration window for the same auditory and visual streams as measured in a previous study (Su, 2014b).

In this task, participants were instructed to attend to both the auditory and the visual sequences while focusing more on the auditory one, as it was task relevant. In half of all the trials, a temporal perturbation could occur in the auditory sequence, such that one of the five auditory downbeats could be delayed or advanced (with equal probability) by 6% of the inter-downbeat interval (i.e., 36 ms). The perturbation could occur either on the second, the third, or the fourth downbeat, with equal probability. Participants were required to respond in each trial whether or not there was any temporal irregularity in the auditory sequence (Figure 1). They were informed that the deviant could only occur in one of the downbeat tones (the “heavier” tones), and never in the upbeat tones. Participants gave their response by pressing one of the two predefined keys. Participants were also informed that the PLF could be moving either downwards or upwards to the downbeats on different trials, and that this was irrelevant to the requested task. To ensure visual attention, in each trial following the response of auditory deviation detection, participants were also asked to recall whether the auditory and visual streams were synchronous or not by pressing one of the two predefined keys (different keys from those for the detection task). They were instructed to base their SJ solely on the subjective impression; it was also stressed that auditory deviation detection was the more important task that should be prioritized, whereas SJ was secondary. This instruction was imposed to avoid compromising the performance of the detection task, which was the primary task of interest.

Each participant underwent five practice trials before starting the experiment. The experiment followed a 2 (AV congruency) × 2 (AV synchrony) × 2 (auditory perturbation) within-participant design, each with 36 repetitions. The total trials were assigned to three experimental blocks of 96 trials each. All the experimental conditions, including the position of auditory perturbation and the nature of perturbation, were balanced across blocks. Within each block the conditions were presented in a randomized order. The entire experiment lasted around 1 h, completed in a single session. A break was required after each block of around 15 min.

### Pilot experiment

It should be noted that the asynchronous AV condition in the present task was implemented by delaying, but *not* advancing, the visual stream relative to the auditory one. This manipulation was based on the result of a pilot experiment, which examined whether the relation between the visual movement phase and the auditory beat was consistently perceived across all the AV combinations. In the pilot experiment, AV synchronous, visual leading (by 150 ms), and visual lagging (by 150 ms) conditions were combined with AV congruent and incongruent presentations as described above, with ten trial repetitions per condition presented in a random order. Ten observers responded in each trial whether they perceived the PLF as moving downwards or moving upwards to the auditory downbeat. It was found that perception of movement phase relative to the downbeat was largely consistent when the auditory and visual streams were synchronous: On average 96% and 99% of the response indicated “downwards” and “upwards” for the congruent and incongruent conditions, respectively. The response was also consistent when the visual stream lagged the auditory one, with 94% and 94% of the response on average indicating “downwards” and “upwards” for the congruent and incongruent conditions. By contrast, when the visual stream led the auditory one, it became less clear to the participants whether the PLF was moving downwards or upwards to the downbeat (on average 51% and 62% of the response for the congruent and incongruent conditions). As the present study intended to manipulate the perceived content congruency with regard to how the PLF moved to the beat, only conditions that yielded consistent perception of such were selected for the main experiment, i.e., synchronous auditory and visual streams, and asynchronous streams in which the visual stream lagged the auditory one.

## Results

### Percentage of deviant detection (hit rate)

**Figure 2:**
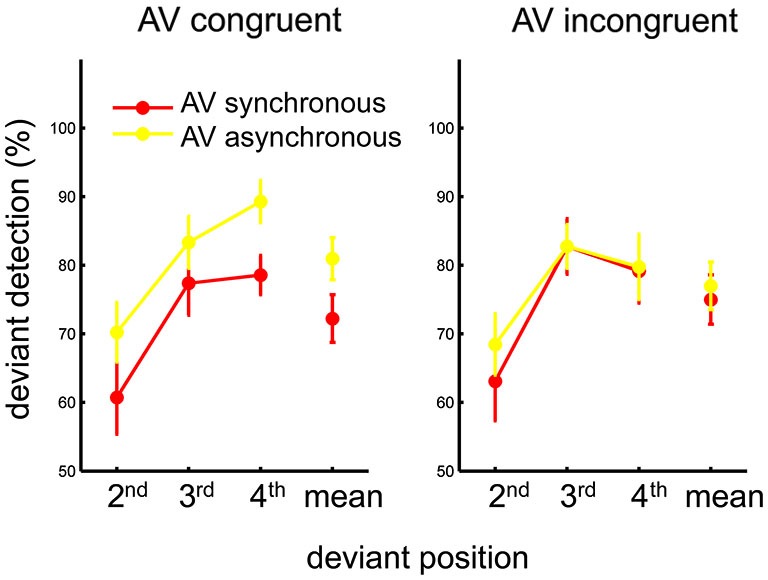
**Mean percentage of auditory deviant detection as a function of the deviant position, for each AV congruency and AV synchrony condition**. The mean across deviant positions for each condition is also plotted in the respective graph. Error bars represent standard error of the means.

Of primary interest was the effect of congruency and synchrony on deviant detection. However, as the present task employed streams of continuous rhythmic stimuli, analyses including the auditory perturbation position as an additional factor may reveal effects related to the predictive nature of the stimuli, as well as its possible interplay with the two main factors. For this purpose, the percentage of correctly detecting an auditory deviant (i.e., the hit rate) for each experimental condition was calculated individually as a first index of the task performance. Individual hit rates were submitted to a 2 (AV congruency) × 2 (AV synchrony) × 3 (auditory perturbation position) within-subject ANOVA. A main effect of synchrony was found, *F*_(1,13)_ = 17.17, *p* < 0.002, *η*^2^ = 0.57, showing a greater hit rate when the AV streams were *a*synchronous than when they were synchronous. A main effect of position was also found, *F*_(2,26)_ = 26.53, *p* < 0.001, *η*^2^ = 0.67. *Post-hoc* tests (Tukey HSD) revealed better detection when the perturbation occurred in the third or fourth beat than in the second beat of the auditory sequence, both *p*s < 0.001. The three-way interaction was not significant, *p* > 0.7. The two-way interaction between congruency and synchrony was significant, *F*_(1,13)_ = 6.12, *p* < 0.03, *η*^2^ = 0.32. As perturbation position did not yield an interaction with any of the two other factors, within-subject means were computed across all positions and submitted to the follow-up one-way ANOVAs conducted for each congruency condition separately. Hit rate was found higher for asynchronous than for synchronous AV streams when they were congruent (i.e., PLF bounced downward to the beat), *F*_(1,13)_ = 17.0, *p* < 0.002, *η*^2^ = 0.57. By contrast, no effect of synchrony was observed when the AV streams were incongruent (i.e., PLF bounced upward to the beat), *p* > 0.2 (Figure 2). Thus, the effect of synchrony on hit rate—i.e., more hits for asynchronous than for synchronous streams—appeared mostly driven by the AV congruent condition.

### Sensitivity (d’)

**Figure 3:**
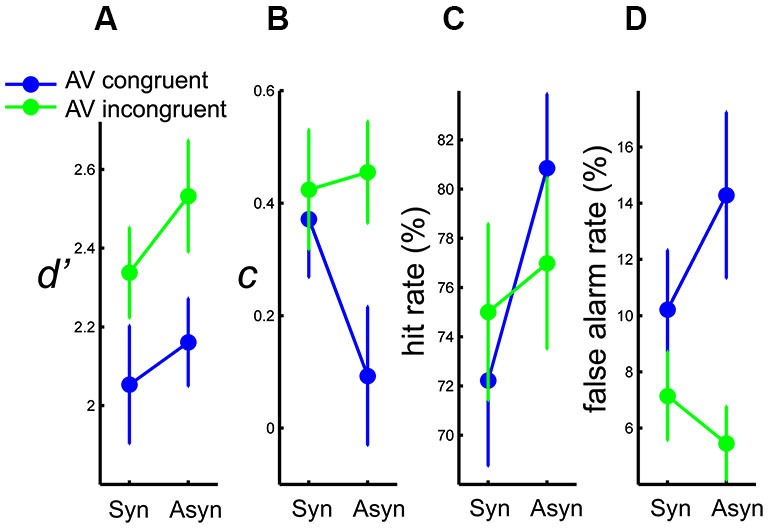
**Results of (A) mean *d’*, (B) mean *c*, (C) mean hit rate, and (D) mean false alarm rate, as a function of AV synchrony, for each AV congruency condition**. Error bars represent standard error of the means.

To assess perceptual sensitivity to the auditory deviants, *d’* was calculated following signal detection theory analysis (“SDT”, Stanislaw and Todorov, 1999) individually for each of the four experimental conditions based on congruency and synchrony. *d’* was calculated as the z-score transformed hit rate minus the z-score transformed false alarm rate. The within-subject *d’*s were submitted to a 2 (AV congruency) × 2 (AV synchrony) repeated-measures ANOVA. A main effect of congruency was found, *F*_(1,13)_ = 5.17, *p* < 0.05, *η*^2^ = 0.28, showing a greater *d’* in the incongruent than in the congruent condition. The effect of synchrony was marginally significant, *p* = 0.07, with a trend of greater *d’* in asynchronous than in synchronous conditions. The interaction between congruency and synchrony was not significant, *p* > 0.6 (Figure 3A). In short, participants were less sensitive to a deviant auditory beat when the observed PLF moved downwards than when it moved upwards to the beat. To some extent, sensitivity to an auditory deviant also seemed lower when the auditory and visual streams were synchronous than when they were asynchronous.

### Response criterion (c)

To examine whether synchrony and congruency also affected processes in the decisional level, the response criterion (*c*) as defined by SDT (averaging the z-score transformed hit rate and the z-score transformed false alarm rate, then multiplied by minus one) was calculated individually for each experimental condition, and submitted to the 2 (AV congruency) × 2 (AV synchrony) within-subject ANOVA. A significant main effect of synchrony was found, *F*_(1,13)_ = 8.21, *p* < 0.02, *η*^2^ = 0.39, showing that participants were more liberal with their response in the asynchronous than in the synchronous condition. The interaction between congruency and synchrony was also significant, *F*_(1,13)_ = 5.59, *p* < 0.04, *η*^2^ = 0.30. Follow-up one-way ANOVAs revealed that the difference in response criterion between synchronous and asynchronous conditions was only evident when the AV streams were congruent, *F*_(1,13)_ = 9.19, *p* < 0.01, *η*^2^ = 0.41, whereas no such difference was found for incongruent AV streams, *p* > 0.6 (Figure 3B). On average, the response criterion as indexed by *c* was positive in all the experimental conditions, showing that participants in this task tended overall to be more conservative than neutral. Participants were more liberal in the asynchronous than in the synchronous condition, but only when observing the PLF moving downwards to the auditory beat.

### False alarm rate

Following the main effect and interaction found in the response criterion, false alarm rates were analyzed to reveal how synchrony and congruency affected the error behavior. (See Section Percentage of Deviant Detection (Hit Rate) for results of hit rate analysis. Results of hit rates were re-plotted here as Figure 3C for better visualization.) Individual false alarm rates were submitted to a 2 (AV congruency) × 2 (AV synchrony) within-subject ANOVA. Only a main effect of congruency was found, *F*_(1,13)_ = 6.09, *p* < 0.05, *η*^2^ = 0.32, showing a higher false alarm rate in the congruent than in the incongruent condition. The interaction between congruency and synchrony was marginally significant, *p* = 0.08 (Figure 3D). As shown, there were generally more false alarms when the PLF moved congruently to the auditory beat. From the marginally significant interaction and the trend of the mean data, it would seem as if participants tended to make more false alarms in the asynchronous than in the synchronous condition for congruent streams.

### Perceived synchrony

To explore whether response in the secondary task (SJ) differed across congruency conditions, individual percentages of responding “synchronous” for each of the experimental conditions were also submitted to a 2 (AV congruency) × 2 (AV synchrony) within-subject ANOVA. A main effect of synchrony was found, *F*_(1,13)_ = 12.97, *p* < 0.01, *η*^2^ = 0.50, with on average 81% and 70% of the response being “synchronous” for the experimental synchronous and asynchronous condition, respectively. The interaction between the two factors was close to significant, *F*_(1,13)_ = 4.61, *p* = 0.051, *η*^2^ = 0.26. A trend was observed of a greater difference in perceived synchrony in the congruent condition (on average 80% and 63% of the response was “synchronous” for synchronous and asynchronous stimuli, respectively) than in the incongruent condition (on average 81% and 77%).

### Relation between each index and the perceived synchrony

Although there were only two objective levels of implemented synchrony (i.e., synchronous or asynchronous), the degree of subjectively perceived synchrony across these two levels may differ amongst individuals (c.f. Su, 2014b). Thus, it was of interest whether and how each dependent variable was related to the extent of perceived synchrony, and whether this relation was varied by AV congruency. To this end, correlational analyses (Pearson’s correlation) were carried out on an individual level (*N* = 14), for the AV congruent and AV incongruent conditions separately, between the following two measures: (1) the difference in the percentage of synchrony response (i.e., the response being “synchronous”) between AV synchronous and AV asynchronous conditions; and (2) the difference in each of the indexes reported thus far (i.e., *d’*, *c*, hit rate, and false alarm rate) between AV synchronous and AV asynchronous conditions.

**Figure 4:**
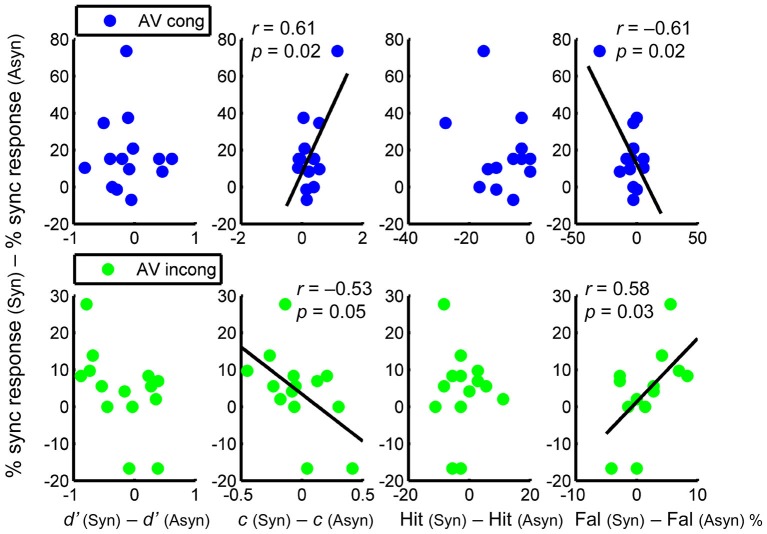
**Relationship between the difference in perceived synchrony and the difference in each parameter (calculated as the difference between AV synchronous and asynchronous conditions), for AV congruent (upper panel) and AV incongruent (lower panel) condition separately**. Columns from left to right: *d’*, *c*, hit rate, and false alarm rate. Pairwise correlations are only significant for *c* and for false alarm rate.

Results revealed significant correlations only in *c* and in false alarm rate, but not in *d’* or hit rate (Figure 4). Regarding *c*, a positive correlation was found in the AV congruent condition, *r* = 0.61, *p* = 0.02, showing that a greater shift to conservative response was associated with a greater increase in perceived synchrony. In the AV incongruent condition, by contrast, the correlation was negative, *r* = −0.53, *p* = 0.05, showing that a greater shift to liberal response was associated with a greater increase in perceived synchrony (Figure 4, 2nd column). As for the false alarm rate, which accounted for the correlations found in *c*, a negative correlation was found in the AV congruent condition, *r* = −0.61, *p* = 0.02, showing that a greater *reduction* in false alarms was associated with a greater increase in perceived synchrony. In the AV incongruent condition, a positive correlation was found, *r* = 0.58, *p* = 0.03, showing that a greater *increase* in false alarms was associated with a greater increase in perceived synchrony (Figure 4, 4th column)^2^. In sum, the difference in response criterion and that in false alarm rate were each correlated with the difference in subjectively perceived synchrony of the AV streams. This correlation, critically, exhibited opposite patterns between congruent and incongruent AV conditions.

## Discussion

The present study investigated how content congruency and temporal synchrony between concurrent rhythmic auditory and visual streams influenced AV integration, as indicated by auditory deviant perception. Participants attended to AV stimuli consisting of a PLF moving regularly to a sequence of auditory beat, and detected a possible auditory temporal deviant. The PLF could move congruently (downwards) or incongruently (upwards) to the beat, while in both situations the movement could be either synchronous with the beat, or lagging behind it. The main results show that, as evidenced by *d’* (Figure 3A), participants were better at detecting an auditory deviant when the PLF moved *in*congruently than congruently to the beat, suggesting stronger integration—or greater visual temporal capture of the auditory beat—in the latter. Similarly, a trend can be noted of stronger visual capture (i.e., lower *d’* for auditory deviant detection) for synchronous than for asynchronous AV streams. Thus, both content congruency and (to some extent) temporal synchrony appeared to promote AV integration in the present scenario.

Specific to the congruent AV stimuli, more hits as well as more false alarms were observed with *a*synchronous than with synchronous AV streams. This synchrony-dependent difference was not seen when the PLF moved incongruently to the beat (Figures 3C,D). Although the increased hit rate in the asynchronous and congruent condition could have been associated with better deviant detection due to lower cross-modal binding, this explanation is challenged by the shift of response criterion (to be more liberal) in this particular condition (Figure 3B), as well as the lack of a corresponding interaction between congruency and synchrony in *d’*. As such, and given the corroborating pattern in false alarms (albeit only marginally significant), this result may rather be explained by the error behavior: namely, more false positives and an increased tendency to report a deviant in asynchronous than in synchronous streams for congruent stimuli. Moreover, congruency also modulated how individual errors were associated with subjectively perceived synchrony: When the PLF moved congruently/incongruently to the beat, a greater increase in perceived synchrony was associated with a greater reduction/increase in false alarms (Figure 4). Thus, in the present task, errors of false alarm were modulated by an interaction between congruency and synchrony. Possible mechanisms underlying these errors will be discussed in Section Content Congruency Modulates Synchrony Effect.

### Cross-modal attention is associated with integration

Owing to the rhythmic nature of both sensory stimuli, the present paradigm afforded the possibility of auditory temporal prediction for the auditory task, which would have rendered the result largely independent of the visual conditions. However, effects of visual manipulation were evident, suggesting that concurrent visual movement information was readily integrated with the auditory rhythm in perception (Su, 2014c). This supports the idea that when multisensory information is available and associated with each other (Lee and Noppeney, 2014; van Atteveldt et al., 2014), cross-modal rather than within-modal attention dominates temporal prediction in each stream, even if the latter alone would have sufficed for the task. Cross-modal prediction has often been shown to underlie perception of AV stimuli that are causally bound in an action, such as AV speech (Zion Golumbic et al., 2013) or AV drumming movements (Petrini et al., 2009b; see also Vroomen and Stekelenburg, 2010, for similar results of artificial visual motion paired with an impact-like sound). Importantly, here it shows that this prediction mode also applies to AV stimuli that are related to each other by means of action-perception coupling (Prinz, 1997), such as observed movements coordinated with external sounds (as in the example of observing dancers moving to music). In this case, the visual movement information is associated with the auditory stream due to the observer’s understanding, or internal representation, of how humans move to rhythmic sounds. For such bimodal rhythmic stimuli, the (possibly obligatory) visual prediction of auditory stream may facilitate coupling between cortical oscillations entrained to each stream, which in turn supports AV integration (Senkowski et al., 2008; Schroeder and Lakatos, 2009).

Depending on the temporal relation and the content match between modalities, effects on AV integration were reflected in how strongly the visual stream attracted an auditory deviant temporally, making it less distinct in some conditions than in others. Such temporal binding of cross-modal stimuli, typically known as “temporal ventriloquism”, has mainly been reported as auditory event(s) shifting the perceived visual onset(s), and not the other way around (Fendrich and Corballis, 2001; Morein-Zamir et al., 2003; Recanzone, 2003). The same modality asymmetry in temporal capture has also been shown in a rhythmic context: Finger taps synchronized to an isochronous visual flashes are considerably attracted to a concurrent but phase-shifted auditory sequence, whereas taps synchronized to tones were rather uninfluenced by concurrent visual distractors (Aschersleben and Bertelson, 2003; Repp and Penel, 2004). The direction of this capture is often taken as evidence of superior temporal processing in the auditory compared to the visual modality (Welch and Warren, 1980). However, recent studies demonstrate that visual rhythm perception and synchronization is much improved when the visual stimulus consists of spatiotemporal periodicity, such as communicated by a moving object (Grahn, 2012a; Hove et al., 2013a,b). Furthermore, previous works have revealed that the same periodic PLF movement as a visual stimulus can modulate auditory rhythm perception (Su, 2014a) as well as improve auditory synchronization (Su, 2014c), and the behavioral gain in the latter study is suggestive of multisensory integration. In this light, the present study presents a new case of visual capture of auditory event in the temporal domain, using visual stimuli derived from biological motion. The integration effect likely originates from perceptual binding of AV information, which occurs when observing a rhythmic human movement while listening to an auditory rhythm (Su, 2014c). Specifically, auditory rhythm perception entails internal motor representation of the rhythm in the listener (Repp and Su, 2013; see also Grahn (2012b), for a review of cortical and sub-cortical motor areas involved in this process). Likewise, observing a human movement elicits internal motor representation (or simulation) of the action in the observer (Jeannerod, 2001). An association between auditory rhythm and rhythmic visual movement that leads to AV binding is proposed to be based on such internal sensorimotor coupling (see Su, 2014c for more relevant discussions).

### Content congruency modulates audiovisual integration

The main findings of the present study are twofold: Multisensory integration was modulated by AV content congruency, as well as by an interaction between AV congruency and AV synchrony. Congruency affected auditory deviant detection, whereas the interaction between congruency and synchrony modulated false alarms and response criterion. Given the effects on these parameters, AV congruency and synchrony appear to modulate integration in both the perceptual and the decisional processes (Meyer and Wuerger, 2001; Wuerger et al., 2003; Sanabria et al., 2007).

Perceptual effects as indexed by *d’* are most consistently associated with congruency, i.e., lower sensitivity to a deviant (indicating greater AV integration) for congruent than for incongruent stimuli. This result is straightforward, and it confirms that cognitive factors such as perceived content match promote integration, as previously shown in AV speech or drumming actions using a SJ or TOJ task (Petrini et al., 2009a; van Wassenhove et al., 2007; Vatakis and Spence, 2007). Notably, congruency can be derived from various forms of AV correspondence (Parise and Spence, 2009; Spence, 2011), and stimuli of abstract correspondences are shown to be processed cortically in a manner similar to multisensory integration (Bien et al., 2012). In this light, the present result reveals a new congruency effect based on whether an observed movement matches an individual’s own motor repertoire coordinated with an auditory beat, i.e., whether it matches how one would naturally move to a beat (see also Su (2014b)). An observed downward movement appears to be favored for integration with an auditory downbeat, compared to an upward movement.

To some extent, synchronous AV streams seem to be associated with stronger visual capture (as indicated by poorer detection) of an auditor temporal deviant, compared to asynchronous ones. This trend is consistent with a large body of literature on inter-sensory binding (Chen and Vroomen, 2013), showing that temporal alignment between the two streams may direct attention in a converging manner to facilitate integration. This pattern is also consistent with the role of cross-modal prediction in multisensory integration (Zion Golumbic et al., 2013; Lee and Noppeney, 2014; van Atteveldt et al., 2014), as visual information in the present asynchronous condition (i.e., visual stream lagging the auditory one) is of little predictive value for the auditory system, thus leading to less integration than in the synchronous condition. However, while some studies show that AV synchrony is critical for auditory enhancement of visual biological motion detection (Saygin et al., 2008; Arrighi et al., 2009), others fail to find support for its importance (Thomas and Shiffrar, 2013). The currently mixed findings may be associated with differences in visual stimuli (e.g., a whole-body figure or only part of it) or the required task (e.g., detection of a walker, other temporal aspects of the movement, or of auditory patterns as presently probed). It may also be that, in studies where temporal synchrony does not modulate multisensory perception, the measured effect reflects inter-sensory priming (Noppeney et al., 2008; Chen and Spence, 2010) rather than integration, which can occur without strict temporal co-occurrence.

Regarding possible neural correlates, in the present task, the observed higher-level, cognitive influence on sensory (here, auditory) processes seems in line with neural findings of higher multisensory regions feedback-modulating lower sensory areas in the course of AV integration (Driver and Noesselt, 2008; Musacchia and Schroeder, 2009). Specifically, the effect of AV congruency is consistent with evidence that neuronal processing in cortical unisensory areas is enhanced by congruent multisensory stimuli, but much less so by incongruent ones (Kayser et al., 2010). Such top-down modulations may be achieved through cortical oscillations between higher-level and lower-level areas (Senkowski et al., 2008; Klemen and Chambers, 2012). In agreement with that, cortical oscillations underlying multisensory integration is also modulated by congruency between dynamic AV stimuli (Gleiss and Kayser, 2014). Finally, semantically congruent and incongruent AV stimuli are often found to engage different cortical multisensory areas, i.e., temporal and inferior frontal regions, respectively (Hein et al., 2007; Doehrmann and Naumer, 2008; van Atteveldt et al., 2010). This pattern is proposed to reflect well-learned associations, or multisensory objects, represented in the temporal regions (e.g., superior temporal sulcus), and conflict monitoring in the inferior frontal areas. It remains to be tested whether the presently proposed top-down influence on lower sensory processes may originate from different multisensory regions depending on content congruency.

### Content congruency modulates synchrony effect

Effects pertaining to post-perceptual, decisional processes were reflected in false alarms and the shift of response criterion. First, there were more false alarms with congruent than with incongruent stimuli. Although it is not entirely clear why, one speculation is that the perceived auditory timing might have been shifted by a mixture of position and velocity cues in the continuous PLF movement trajectory (Su, 2014b). This shift was likely not stable or constant for all the auditory events, causing occasional fluctuation in the perceived auditory onsets and thus erroneous judgment of a deviant. The observed effect was greater for congruent than for incongruent stimuli, arguing for stronger AV binding in the former. Next, in particular, a curious pattern of increased false alarms and more liberal response was seen in *a*synchronous streams, and the effect was mainly evident when the observed movement was congruent with the auditory rhythm. One plausible explanation for this pattern, paradoxically, also rests upon visual temporal capture of auditory beats: In an asynchronous AV situation, the auditory events might be temporally shifted by the visual stream due to AV binding (i.e., temporal ventriloquism). If, as suspected, this shift is occasional and not constant throughout the auditory sequence, the perceived irregularity could be erroneously taken as a deviant, leading to a false positive response. Notably, this effect is specific to the congruent AV stimuli, suggesting that content congruency can promote (potentially erroneous) integration of AV information at greater temporal distance. As such, the effect of temporal proximity as a low-level stimulus factor on integration seems to be modulated by higher-level cognitive factors, such as the perceived content match. One question, then, is whether this result pattern might be associated with the observation that the difference in perceived synchrony between synchronous and asynchronous conditions (as measured in the secondary task) seems greater in congruent than in incongruent stimuli. Put in another word, is subjective AV asynchrony directly linked to the auditory susceptibility to visual temporal capture? There seems to be evidence against this speculation (Stevenson et al., 2012): A narrower AV temporal integration window (i.e., lower tendency to perceive asynchronous stimuli as synchronous) is correlated with a *lower* tendency to integrate asynchronous stimuli, and thus—in the present case—it should have led to fewer, and not more, false alarms.

A similar interaction between stimulus timing and content congruency has been described in a recent study of AV speech (syllable) perception (Ten Oever et al., 2013), in which semantically congruent AV stimuli compared to incongruent ones are integrated at greater temporal disparity. This leads to the proposal that, as opposed to lower-level stimulus features (e.g., timing) and higher-level cognitive factors (e.g., semantic congruency) operating serially and hierarchically, these two factors may in fact work in parallel to reach a perceptual outcome (Stevenson et al., 2014b). In line with this proposal, the present results extend the principle to a non-speech action domain involving continuous AV stimuli, whose congruency is derived from internal motor simulation (Jeannerod, 2001). It may be argued that such top-down cognitive mechanisms, based on sensorimotor coupling, operate in parallel with bottom-up, synchrony-driven attention (Van der Burg et al., 2008; Fiebelkorn et al., 2010) in the course of multisensory integration of rhythmic stimuli.

Also regarding the interaction between the two factors, under congruent conditions, a greater increase in perceived synchrony was associated with a greater *decrease* in false alarms across individuals. Under incongruent conditions, however, a greater increase in perceived synchrony was associated with a greater *increase* in false alarms. These patterns may be explained in terms of individual differences in AV synchrony perception predisposing the strength of AV binding (Stevenson et al., 2012), and this tendency leads to different consequences of error, depending on content congruency. With congruent content, the more an individual is able to discern synchronous from asynchronous streams, the more the streams may be unambiguously integrated in the former and less in the latter, thus reducing the chance of visual capture of asynchronous auditory stimuli and the subsequent false alarms. By contrast, incongruent content may increase uncertainty in synchronous situations, possibly due to conflicting information regarding the unity of stimuli (Welch and Warren, 1980), i.e., the incompatible movement relative to the beat deters the perceptual system from integration, whereas synchrony between the streams promotes it. As a result, individuals who can better tell apart synchronous from asynchronous situations are subject to greater perceptual conflict, leading to more errors.

The interaction between content congruency and temporal synchrony seems to occur later in the decisional stage (as reflected in the response criterion) compared to its perceptual effect (as reflected in sensitivity). From the literature, congruency seems to modulate the time course of multisensory processing, with a larger early response to congruent (compared to incongruent) stimuli (Naci et al., 2012), followed by a later response to incongruent (compared to congruent) ones (Meyer et al., 2013). Although it remains speculative at present, it is possible that an earlier feedback modulation through congruent AV stimuli (Naci et al., 2012) would contribute to temporal capture or integration in the auditory cortices (Musacchia and Schroeder, 2009; Marchant and Driver, 2013), whereas feedback from incongruent stimuli may occur later and, rather than interacting with stimulus timing for integration, it would be more involved in conflict resolution.

Finally, it is worth mentioning that in the pilot experiment, an asymmetry was evident regarding how AV temporal order influenced the perceived movement direction relative to the beat: Judgment of direction (implying congruency) was more ambiguous when the visual stream led—compared to when it lagged—the auditory one. Together with the observation in the main experiment that congruency appeared to influence perceived synchrony, it is possible that AV temporal relation and content congruency in the present scenario interact with each other both-ways in perception. Although a detailed discussion on this point is beyond the scope of the present research, future investigations using different paradigms are warranted to gather further evidence of this interaction, and its implication in multisensory perception.

### Multisensory integration vs. attentional entrainment in rhythmic stimuli

In the domain of multisensory integration, attention is often considered to be a mechanism that can facilitate cross-modal binding (Fiebelkorn et al., 2010; Koelewijn et al., 2010; Talsma et al., 2010). There is, however, a different framework pertaining to the role of attention, namely that of the *Dynamic Attending Theory* (“DAT”, Jones and Boltz, 1989; Large and Jones, 1999) as briefly mentioned in the Introduction, which is also relevant in the context of bimodal rhythmic stimuli. Discussions are thus warranted as to possible overlaps and discrepancies between DAT and theories of integration when explaining multisensory perception. DAT proposes that attention can be seen as an oscillatory energy, and it is temporally entrained by the periodicity of the external sensory rhythms, leading to enhanced stimulus processing at the expected points in time. Findings in support of this theory typically show that a deviant is better detected when its expected occurrence coincides with the entrained periodicity (Jones et al., 2002, 2006; Repp, 2010; Su, 2014a). This model is further corroborated by possible neural correlates, such as cortical oscillations in the beta band being phase-locked to a regular auditory beat (Large and Snyder, 2009; Iversen et al., 2009; Fujioka et al., 2012). Within this framework, synchronous multisensory rhythms compared to asynchronous ones are expected to facilitate such processing by entraining attention to convergent points in time (Nozaradan et al., 2012; Su, 2014c). As such, in the present case, DAT would predict that synchronous AV streams should yield better auditory deviation detection than asynchronous ones, while content congruency should not play a critical role. These predictions run contrary to those made with regard to AV integration and inter-sensory capture. At first sight, the present results seem to support the latter.

Can these two accounts—thus far situated in somewhat different research domains and yet both tapping onto the operation of attention—be reconciled in addressing multisensory perception of rhythmic stimuli? Inspection of the present data suggests that these two accounts may be combined to explain the results. First, deviants were better detected in later temporal positions, which appears to reflect the effect of attentional entrainment, as expectation can be more strongly and precisely generated with more repetitions of intervals preceding a possible deviant (Haenschel et al., 2005). This effect was independent of AV congruency and synchrony, i.e., the mechanism exists independently of the concurrent visual information, suggesting that it functions as a perceptual basis for rhythmic stimuli at least in the task-relevant modality. On top of that, auditory deviant detection varied according to AV congruency and to some extent synchrony, and the effect was consistent with predictions of AV integration rather than of bimodal entrainment alone. Based on these results, the present research proposes the following: In the context of multisensory rhythms, attention is temporally entrained by the (especially task-relevant) stimulus rhythmicity, likely in a bottom-up, automatic manner (Bolger et al., 2013). This temporal orienting serves a general perceptual frame for stimulus processing that is less sensitive to specificities of multisensory information. Indeed, literature on attentional entrainment consistently shows that enhanced attention can be flexibly transferred across modalities and tasks (Escoffier et al., 2010; Bolger et al., 2013; Brochard et al., 2013). However, owing to the heightened attention entrained by the stimulus rhythmicity, multisensory binding around these points in time is also enhanced, which is then subject to modulations of variables critical for integration, such as congruency and synchrony. Presently it would seem as if the same attentional capacity is deployed for temporal entrainment and multisensory integration in a hierarchical manner, with the former serving the basis for the latter.

There seems to be a link between the DAT model and multisensory integration: With respect to attentional entrainment, a body of neurophysiological research demonstrates that rhythmic cortical oscillations can be entrained (i.e., the neuronal excitatory phase being aligned) to the rhythmicity of external stimuli, such that neuronal responses to the sensory input are amplified (e.g., Lakatos et al., 2008; Schroeder and Lakatos, 2009). This operation is especially instantiated by deploying attention to the task-relevant stimulus stream amongst other phase-shifted streams in a different modality (Lakatos et al., 2008, 2013). Most critical in the context of multisensory stimuli are proposals that oscillations in one lower sensory area can be phase-reset, in a predictive manner, by concurrent input from another modality (Lakatos et al., 2007; Schroeder et al., 2008), a mechanism that is argued to underlie multisensory integration (van Atteveldt et al., 2014). These findings seem to support the hypothesis proposed above. Namely, the task-relevant rhythmic stream entrains internal processes (i.e., oscillations) in the temporal domain through attentional deployment, while input from another modality—dependent upon multisensory correspondence—modulates the processes on top of this entrainment, thus enhancing or impeding integration. From here on, other relevant hypotheses can be tested, e.g., in rhythmic stimuli comprising several hierarchical levels of periodicity, whether the strength of integration would vary according to the saliency (and thus potential for entrainment) of each periodicity.

In conclusion, the present study highlights the effect of the cognitive factor (content congruency), as well as its interaction with the stimulus factor (temporal synchrony), on integration of continuous, rhythmic AV information related to human movements and extraneous sounds. A new form of congruency is demonstrated here, based on whether the observed movement matches how humans typically move to an auditory beat (i.e., action-perception coupling). This content congruency influences integration, as well as whether attention may be spread despite inter-sensory asynchrony to support integration. Consistent with previous findings in AV speech, perception of complex AV actions may also entail parallel processing of lower-level stimulus parameters and higher-level content correspondence. As a multitude of environmental and biological signals are multisensory and rhythmic (Arnal and Giraud, 2012), possible interplays amongst factors of integration and rhythm perception remain an interesting scenario for further explorations.

## Conflict of interest statement

The author declares that the research was conducted in the absence of any commercial or financial relationships that could be construed as a potential conflict of interest.

## Footnotes

^1^As noted in Su (2014a), this constituted a departure from the original nature of a point-light display, where the figure motion is perceived from unconnected moving discs (Blake and Shiffrar, 2007).

^2^Although the correlations found in the AV congruent conditions might have been driven by one extreme data point, there is no apparent reason (e.g., experimental errors) to consider this data point illegitimate for inclusion. In the presence of a potential outlier, correlation analyses were performed again on square root transformed data (Osborne, 2002). While the correlation found in the AV congruent condition was not significant any more for c (*r* = 0.46, *p* = 0.1), the negative correlation remained significant for the false alarm rate (*r* = −0.56, *p* = 0.03).
